# Intra-articularly injected mesenchymal stem cells promote cartilage regeneration, but do not permanently engraft in distant organs

**DOI:** 10.1038/s41598-019-46554-5

**Published:** 2019-07-12

**Authors:** María Satué, Christiane Schüler, Nikole Ginner, Reinhold G. Erben

**Affiliations:** Department of Biomedical Sciences, University of Veterinary Medicine, Vienna, Austria

**Keywords:** Mesenchymal stem cells, Mesenchymal stem cells

## Abstract

Intra-articular (IA) injection of mesenchymal stem cells (MSCs) promotes articular cartilage repair. However, cell fate and action after transplantation remain unclear. This study aimed at evaluating the biodistribution and efficacy of MSCs after IA injection. We used an immunocompetent, dual transgenic rat model, which is based on donor rats ubiquitously expressing heat stable human placental alkaline phosphatase (ALPP), and recipient rats expressing a heat sensitive ALPP form. A focal cartilage defect was created in the patellofemoral groove of recipient rats. Bone marrow-derived MSCs isolated from donor rats were injected into the synovial cavity of recipients, and cell tracking was performed in distant organs and knees over 6 months post-injection. A few donor MSCs were observed in the lung of one of the recipients, 1 day post-injection. We failed to detect donor MSCs in any of the studied tissues at all later time points. IA-injected MSCs remained in the synovial cavity, engrafted within the cartilage lesion, and were detectable up to 1 month post-injection. Although the number of MSCs decreased over time, MSCs injection promoted cartilage regeneration as evidenced by histology and immunofluorescent collagen staining. Our study supports the safety and efficacy of using MSCs for cartilage repair via IA delivery.

## Introduction

The loss of articular cartilage due to trauma or degeneration requires new clinical strategies to cope with the very limited self-renewal capacity of this tissue^1^. Different techniques including bone marrow stimulation, allografts and autografts have been applied to repair cartilage defects^2^. However, to date, results are not satisfactory as they often result in the development of fibrocartilage with inferior mechanical properties, and in the lack of proper neotissue integration^3^. Mesenchymal stem cells (MSC) are currently a potential therapy for treating a variety of diseases, such as articular cartilage defects. In particular, bone marrow-derived MSCs (BM-MSCs) have a high potential for cartilage repair because of their ready availability and high chondrogenic efficiency^4^. In addition to their differentiation capacity, MSCs are considered environmentally-responsive cells, as they are able to influence their microenvironment by secreting different growth factors, anti-inflammatory mediators, anti-catabolic or immunomodulatory factors^5–7^.

Intra-articular (IA) injection of MSCs is a simple, minimally invasive and efficient delivery procedure. A large number of preclinical studies have proved the beneficial effects of IA delivery of MSCs on cartilage morphology and histology, showing a better tissue repair and less cartilage destruction^8–17^. In accordance with this, a growing number of clinical trials revealed that MSC therapy via IA injection improves pain and functional properties by stimulating cartilage regeneration^18–26^. Thus, IA administration of MSCs into the knee of osteoarthritic patients appears to be effective, reducing the pain and improving articular cartilage regeneration and physical function^27^.

Due to the significant clinical relevance of the application of MSCs for treating tissue damage, there is an urgent need to better characterize the fate and action of MSCs after IA injection. A recent study from our group, using a novel immunocompetent transgenic model, showed that intra-articularly injected MSCs were able to engraft to injured articular cartilage and contributed to healing of a focal cartilage defect^28^. The main advantage of the model is that it mimics the real patient situation, since it permits tracking genetically labeled cells without immune-mediated rejection in a host with intact immune system. Furthermore, our system allows highly reliable MSC tracking through histochemical staining, being a more sensitive and specific method compared to bioluminescent, biofluorescent imaging or real time qPCR analyses. Because of these advantages, we were able to visualize the injected stem cells at the defect site, and showed that they did not remain in the injured cartilage tissue in the long term, suggesting a non-progenitor role of MSCs in cartilage regeneration.

For the development of a safe and effective clinical therapy, it is fundamental to thoroughly assess distribution and migration of intra-articularly injected MSC within the joint and in other distant organs, as well as their mode of action in cartilage regeneration. In this study, we used our dual transgenic rat model to track intra-articularly injected MSCs in the knee joint and in some distant organs of recipient rats with a focal cartilage defect. Homing of injected MSCs in the different organs was investigated by histochemical staining, and inflammatory response and cartilage regeneration was evaluated by immunofluorescence and histochemical analysis.

## Results

### Viability of MSCs after injection

**Figure 1:**
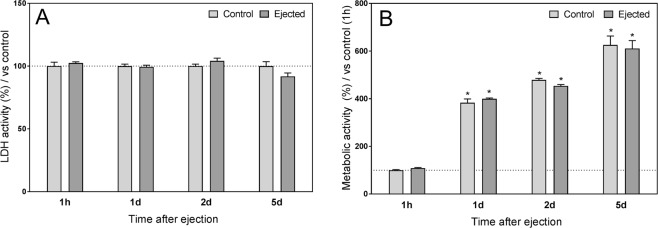
Effect of injection on MSC viability *in vitro*. (**A**) LDH activity was measured at 1 hour, 1 day, 2 days and 5 days after ejection through a 23 G needle. Data were normalized to control for each time point (non-injected cells, 100%). Values represent the mean ± SEM; n = 6. No significant differences were determined between control and injected groups. (**B**) Metabolic activity was measured at 1 hour, 1 day, 2 days and 5 days after ejection. Data were normalized to the 1 hour post-ejection control group (100%). Values represent the mean ± SEM; n = 6. No significant differences were detected between control and injected groups for any of the studied time points. However, metabolic activity increased over time in both groups. *P < 0.05 vs. 1 hour after ejection in the corresponding group by Kruskal-Wallis test followed by Mann-Whitney *U*-test.

**Figure 2:**
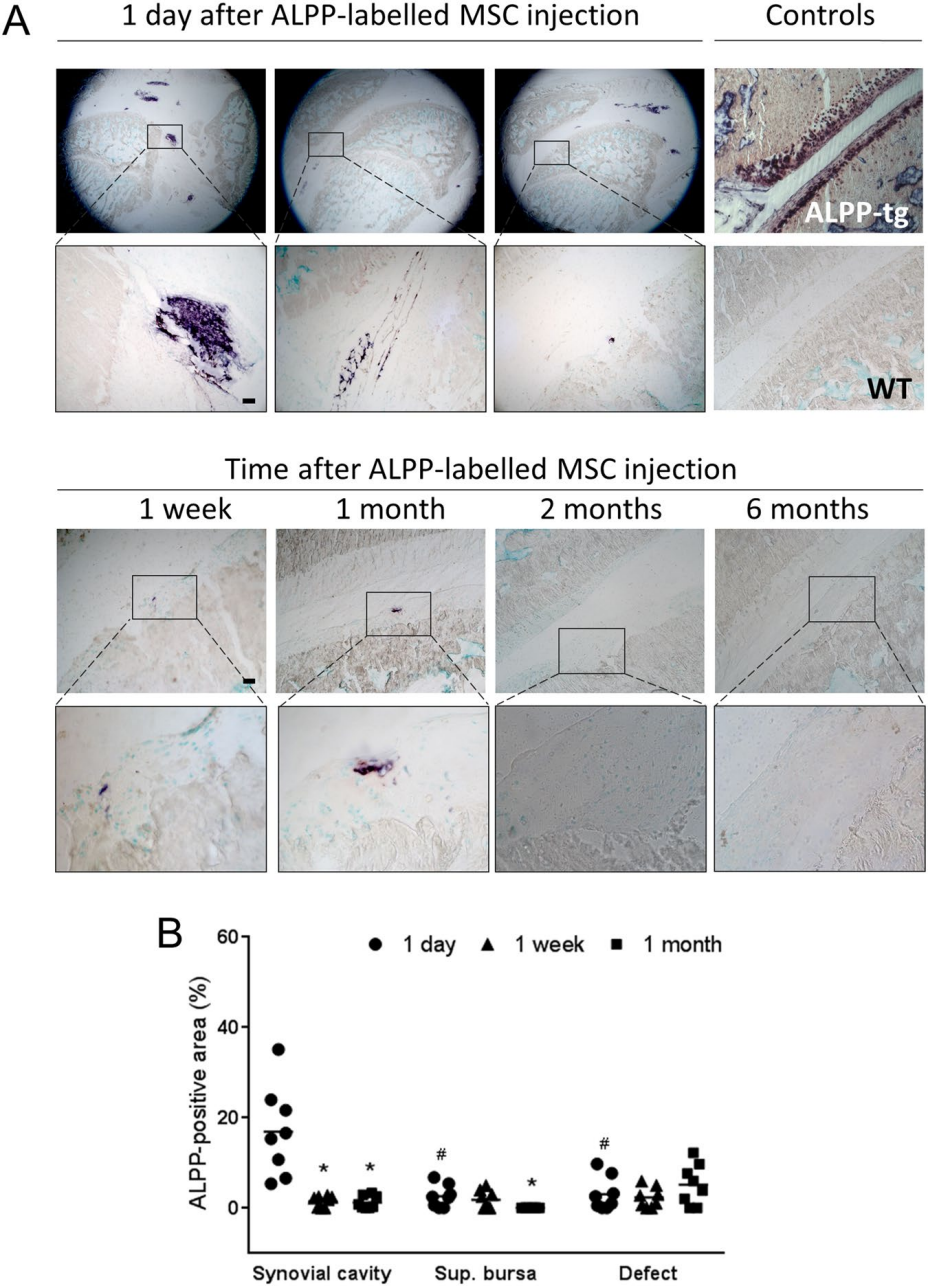
Evaluation of histochemical ALPP staining in cryosections from knees of ALPP-transgenic and WT controls, and from ALPP^m^ knee joints with a focal cartilage defect, 1 day, 1 week, 1 month, 2 months and 6 months after injection of ALPP-labeled MSCs. (**A**) Representative images of histochemical ALPP staining. Insets in the upper panels show ALPP-positive staining in the synovial cavity (left), suprapatellar bursa (middle) and cartilage lesion (right), at 1 day post-injection. Most MSCs were found in the knee joint cavity. ALPP-labeled MSCs were also detected in the synovial cavity and the cartilage defect, 1 week and 1 month after MSC injection (lower panels). No MSCs from ALPP donors were detected at 2 or 6 months after ALPP-labeled MSC injection (lower panels). Scale bar = 100 μm. (**B**) Quantification of the ALPP-positive area in the synovium, suprapatellar bursa (sup. bursa) and defect area. Dots represent percentage of ALPP-positive area; at least 2 sections per animal were analyzed (n = 4 animals per group). *P < 0.05 vs. corresponding region 1 day after injection; ^#^P < 0.05 vs. synovial cavity at the same time point by Kruskal-Wallis test followed by Mann-Whitney *U* test.

In order to assess the effects of injection on cell viability, we tested MSC viability over 5 days after ejection through a 23 G needle *in vitro*. MSCs were maintained in culture, and LDH and PrestoBlue analyses were performed. Measurement of LDH activity, an index of cytotoxicity, showed no differences between the ejected and non-ejected control MSCs (Fig. 1A). Similarly, no significant differences between the two groups were observed in the metabolic activity at the different time points (Fig. 2B). However, the metabolic activity in both control and ejected MSCs increased over time, suggesting that the injection procedure did not change the cells’ proliferating behavior.

### MSCs distribution and engraftment in the knee joint

To analyze distribution and engraftment of intra-articularly injected MSC, we used a full thickness articular cartilage defect model in the patellofemoral groove in the current study. This type of defect exposes the subchondral bone, and leads to the initial formation of a fibrin clot at the injury site. The knee joints from the recipient rats (ALPP^m^) were dissected and histochemically analyzed to study the presence of ALPP-labeled MSCs over time (Fig. 2). We did not observe any adverse effects after intra-articular injection of MSCs or serum. There were no animals excluded from the analyses. As expected, a large number of donor MSCs was observed in the knee joints, 1 day after cell delivery. Furthermore, cells were not homogenously distributed through the knee cavity, but formed cell aggregates suspended in the synovial fluid. A few cells were observed in the defect area (3.1 ± 1.3%), suggesting their intrinsic capacity to migrate rapidly towards the injury site. Nevertheless, most cells remained in the synovial cavity (16.9 ± 3.5%) and some cells were found in the suprapatellar bursa space (2.6 ± 0.9%). One week and 1 month after cell injection, ALPP-labeled MSCs were also detected in the cartilage lesion (2.3% ± 0.8 and 5.1% ± 1.6, respectively) and the synovial cavity (1.5% ± 0.4 and 1.2% ± 0.5, respectively). However, the number of ALPP-labeled MSCs in these localizations was decreasing over time. Similarly, the number of ALPP-labeled MSCs in the suprapatellar bursa also declined with time (1.8% ± 0.8 at 1 week post-injection), and no cells were observed at and beyond 1 month after cell delivery (Fig. 2B). Two and 6 months after MSC injection, ALPP-labeled MSCs were neither present in the cartilage lesion area nor in the knee cavity (Fig. 2A)

### Migration of MSCs to distant organs

**Figure 3:**
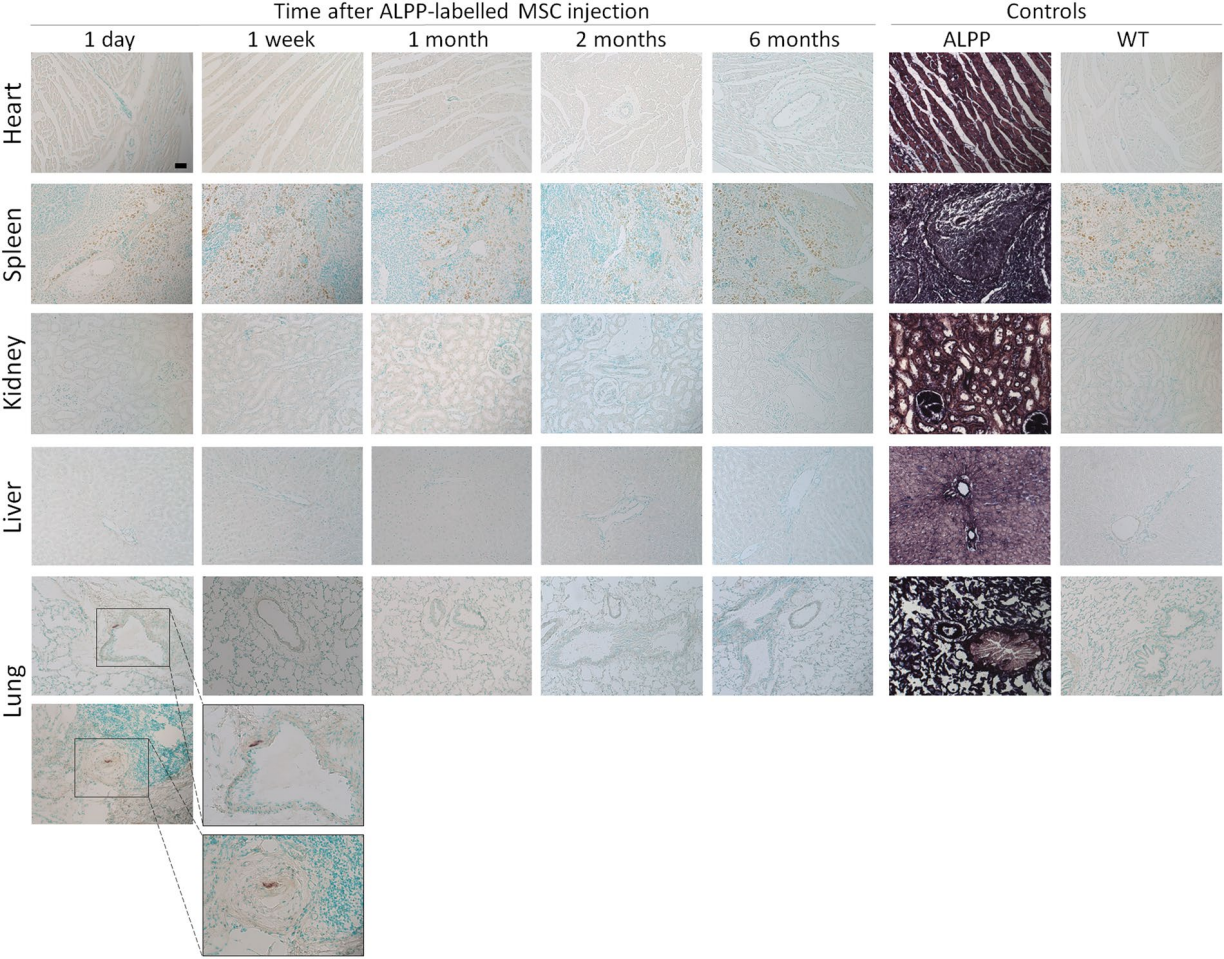
BCIP/NBT staining of paraffin sections from various organs of ALPP-transgenic and WT controls, and of ALPP^m^ recipient rats at 1 day, 1 week, 1 month, 2 months and 6 months after intra-articular injection of ALPP-labeled MSC. No enzyme activity could be detected in heart, spleen, kidney or liver from ALPP^m^ recipients rats intra-articularly injected with MSC from ALPP donors. However, a few ALPP-labeled MSCs were identified in the perivascular space, surrounding pulmonary blood vessels, 1 day after injection, but only in one of the studied animals. ALPP donors show strong staining in all tissues, while no staining is observed in wild-type (WT) controls. Scale bar = 50 μm; n = 4 animals per group.

Different distant organs were collected from the transgenic recipient rats that were intra-articularly injected with donor ALPP-labeled MSCs. Histochemical staining of the different distant organs revealed the presence of ALPP-labeled MSCs in the perivascular space around pulmonary blood vessels in one of the studied animals, 1 day after cell injection (Fig. 3). However, no donor transgenic MSCs were detected in the lung of the recipient rats at later time points (1 week, 1 month, 2 months or 6 months after cell injection). Furthermore, we failed to identify ALPP-labeled MSCs in the heart, spleen, kidney or liver at 1 day, 1 week, 1 month, 2 months and 6 months after cell delivery.

### Therapeutic efficacy of MSCs for cartilage regeneration

**Figure 4:**
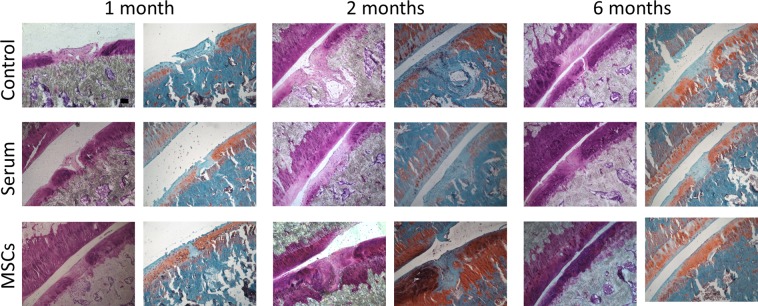
Toluidine Blue and Safranin O staining of cryosections from ALPP^m^ knee joints at 1 month, 2 months and 6 months after injection of ALPP-labeled MSCs. Cartilage regeneration in the injured site was observed in the knee joints that were previously injected with 10^7^ ALPP-labeled MSCs, suspended in rat serum, but not in ALPP^m^ recipients injected with serum or left untreated. Scale bar = 100 μm; n = 4 per group.

To evaluate cartilage regeneration, toluidine blue and safranin O stainings were performed. Representative photomicrographs of the focal articular cartilage defects at 1 month, 2 months, and 6 months after ALPP-labeled MSC injection are shown in Fig. 4. One month after cell injection, tissue similar to neocartilage was observed in the MSC-injected group, whereas unsatisfactory tissue integration and apparent tissue gaps were found in the control and serum groups. Compared with control and serum-injected rats, which showed poor matrix staining as well as abnormal tissue structure, improved matrix staining was observed in the defects, 2 months after MSC injection. Notably, MSCs-treated rats exhibited almost normal hyaline cartilage tissue at the defect site, 6 months after cell injection. Toluidine blue and safranin O staining within the defect site was similar to the surrounding tissue, suggesting the formation of healthy neocartilage. In contrast, both stainings revealed a poor content of proteoglycans in the neotissue matrix in control and serum samples.

**Figure 5:**
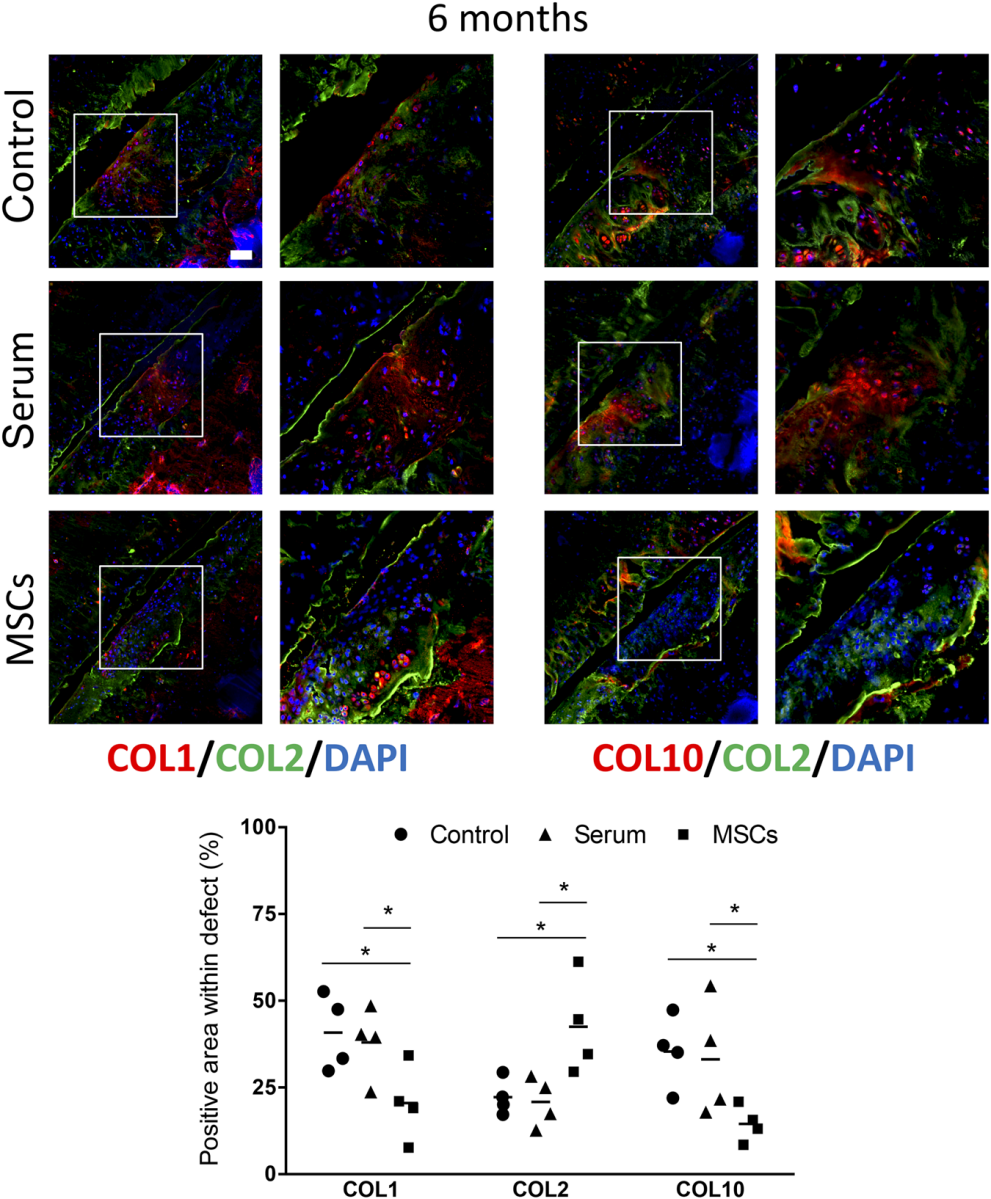
Collagen content in cryosections from ALPP^m^ knee joints at 6 months after injection of ALPP-labeled MSCs. (**A**) Representative immunofluorescence stainings for collagen type-1 (COL1, red), collagen type-II (COL2, green) and collagen type-X (COL10, red) in the cartilage defect areas. Scale bar = 100 μm. (**B**) Quantification of the collagen content in the defect area, 6 months post-injection. Dots represent collagen-stained area within the defect (n = 4 per group). COL1 and COL10 formed the neotissue matrix in control and serum samples, whilst IA-injection of MSCs led to enhanced COL2 and almost absent COL1 and COL10 expression in the focal cartilage lesions. *P < 0.05 by one-way ANOVA followed by Tukey’s multiple comparison test.

Further evaluation of the collagen content at the defect sites was performed by immunofluorescence staining (Fig. 5). Collagen type I (COL1) was highly expressed in the cartilage defects of control and serum-injected rats. Conversely, a lower expression of COL1 was found in defects of MSC-treated animals. Interestingly, collagen type X (COL10), a marker of hypertrophic cartilage, was also highly expressed in the defects in control and serum-injected knees, but was almost undetectable in defects of MSC-injected rats. In contrast, defects from MSC-injected rats showed a higher abundance of collagen type II (COL2), the main component of healthy articular cartilage.

### Evaluation of the inflammatory response after IA injection of MSCs

**Figure 6:**
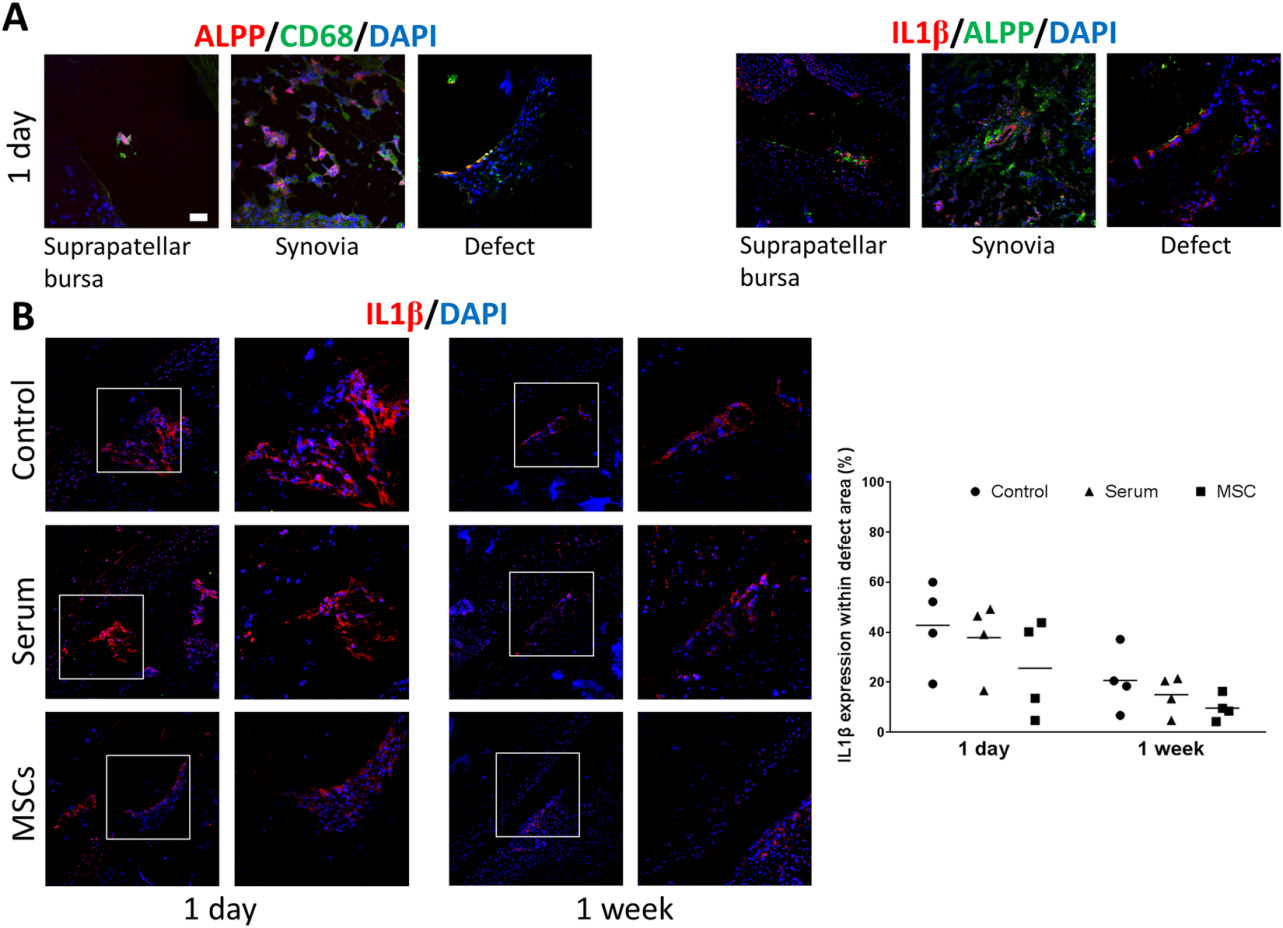
Immunofluorescence staining of cryosections from ALPP^m^ knee joints at 1 day and 1 week after injection of ALPP-labeled MSCs. (**A**) IA-injected ALPP-labeled MSCs were detected surrounding CD68- and interleukin 1β (IL1β)-positive cells in the suprapatellar bursa, synovia and cartilage defect, 1 day after injection. (**B**) Inflammatory response in the defect area was evaluated by means of IL1β protein expression. Data were analyzed using Kruskal-Wallis test followed by Mann-Whitney U-test. A trend to more intense staining was observed in control and serum samples compared with MSC-treated defects, but differences were not statistically significant (P > 0.05). Scale bar = 50 μm; n = 4 per group.

In order to investigate the modulatory effect of MSCs on the inflammatory response resulting from the surgical procedures and the cartilage lesion, we analysed the presence of CD68^+^macrophages and of interleukin 1β (IL1β) by immunofluorescence, 1 day after MSC injection (Fig. 6A). We observed macrophages and IL1β expression in the suprapatellar bursa, synovial tissue and at the defect site. Interestingly, ALPP-labeled MSCs were also located in these regions, and were surrounding CD68- and IL1β-positive stained areas. The IL1β protein expression in the cartilage defects tended to be higher in control and serum-injected rats, compared with MSC-treated animals. However, the differences between the groups were not statistically significant due to a large inter-individual variance (Fig. 6B).

## Discussion

In the present study, we demonstrated the safety and efficacy of IA injection of MSCs for promoting articular cartilage regeneration in a focal defect model. Using an immunocompetent transgenic rat model that allows sensitive histochemical staining, we tracked the injected, genetically labeled cells in the knee joint and in distant organs. To the best of our knowledge, this is the first study evaluating the biodistribution of intra-articularly injected MSC in an unbiased cell tracking model, mimicking the situation in a human patient treated with autologous MSC. Most cells remained in the synovial cavity, although some MSCs migrated and engrafted into the damaged cartilage as early as the first day after injection. At this time point, a few MSCs were observed in the lung of one of the recipient animals, but no cells were detected in distant organs at all later time points. Furthermore, in contrast to the repair mechanisms involving fibrocartilage and bone formation found in the control groups, the neotissue formed at the defect site following MSC injection showed a collagen and matrix composition closely resembling that of normal articular cartilage, 6 months post-injection.

There is increasing evidence that therapeutically administered MSCs can positively influence the regeneration of damaged tissues, such as articular cartilage lesions. However, the fate of the transplanted cells is still under debate. Most studies agree that when MSCs are IA-injected, cells tend to remain at the injection site, because the joint provides a confined space. Lee *et al*.^29^ found MSCs in the neocartilage tissue formed at the defect site, 3 months after IA injection. Similarly, MSCs were detected at 8, but not at 16 weeks after implantation in an osteochondral defect using a hydrogel^30^. A recent report^17^ confirmed that IA-injected MSCs remained in the knee joint for 14 days. In contrast, in another recent study^15^, MSCs were found in the knee cavity and synovial tissue at 1 day after injection, but could not be detected in the knee joint at 1 week post-injection. The discrepancies between the existing studies are probably the result of different experimental settings, such as animal model, cell type and number, labelling techniques, timing of injection and follow-up, or sensitivity of the method employed for cell tracking analysis. In our study, using an immunocompetent animal model that allows sensitive histological cell tracking in the absence of immune-mediated rejection of transplanted cells, we were able to track IA-injected MSCs in the cartilage defects, synovial tissue and supra-patellar bursa for 1 month post-injection, although the cell number decreased over time. In order to find out whether the injection process caused cell damage, cell viability after ejection was determined *in vitro*, but no negative effect was observed. Thus, the rapid disappearance of the injected cells within the joint may be explained by the lack of extracellular matrix for attachment, which might induce anoikis of MSCs^31^. To improve cell survival, mobility and proliferation of MSC in injured microenvironments, genetically engineered MSCs have been employed^32–34^. These lines of research may lead to more efficient MSC-based therapies in the future.

Based on current clinical trial outcomes, therapy with autologous or allogeneic MSCs is safe, with no significant adverse events following IA^35^ or even intravascular injection^36^. Surprisingly, only few pre-clinical studies have evaluated the safety of IA injection of MSCs. Horie *et al*.^37^ failed to detect genetically labeled rat donor MSCs in distant organs of wild-type recipient rats when using luminescent and real-time qPCR analysis. However, a possible confounding factor in the latter experimental system is immune-mediated rejection of MSCs expressing foreign proteins in immunocompetent hosts^38–40^. In contrast, using a PCR-based Alu sequence detection approach, IA-injected human MSCs in SCID mice were observed in bone marrow, adipose tissue and skeletal muscle for the first 3 months post-treatment^41^. Conversely, using the same detection marker (Alu specific qPCR), Shim *et al*.^42^ demonstrated that the blood levels of human MSCs in Balb/c nude mice peaked 8 hours post IA-injection and gradually declined thereafter, but cells were only detected in the joint tissue. As expected, IA-injected human MSCs were not found in any other organs than the knee joint when using immunocompetent mice^43^. Similarly, following IA injection in an immunocompetent rat osteoarthritis model, human MSCs could not be detected by specific hFOXP2 gene analysis in any studied tissue except for the knee joint^12^. Taken together, there is good evidence that IA-injected xenogeneic MSC are rapidly cleared from the rest of the body in immunocompetent hosts, but the fate of IA-injected allogeneic or autologous MSCs is less clear.

In the animal model employed in the current study, donor and recipient rats are of the same inbred strain, and differ only in the transgenic expression of wild-type or mutant ALPP^28^. Hence, this model closely mimics the situation in a patient treated with autologous cells. Using this model, the present work has shown that IA-injected MSC were able to migrate through blood vessels and could be detected in the lung at 1 day post-injection in one recipient rat. It is conceivable that some MSCs may enter blood vessels during the injection procedure, or migrate through the synovia into blood vessels. It is well known that intravenous MSC delivery causes rapid cell entrapment in the lungs^42–44^, with cell retention declining at a very fast rate^42^. Indeed, it was recently found that monocytes rapidly phagocytose infused MSCs^45^. Our finding that some IA-injected MSCs were entrapped in the lung of one recipient rat, 1 day after injection, is in line with these earlier reports. However, we found no evidence of permanent engraftment in any studied organ at all later time points, suggesting a rapid clearance of the migrated MSCs, and supporting the safety of IA therapy with MSCs.

The potential of MSCs for cartilage regeneration is well known. There are many reports in the literature demonstrating the beneficial effect of MSCs for promoting articular cartilage repair^11,13–16,30,46–49^. In our study, IA injection of bone marrow-derived MSCs improved cartilage regeneration at the lesion site, as evidenced by toluidine blue and safranin O staining, low expression of markers specific for fibrocartilage and early bone differentiation such as COL1 and COL10, and high expression of COL2, a marker of healthy articular cartilage^15,16,30,46–49^. Our results suggest that injected MSCs inhibited fibrotic and hypertrophic remodelling, whilst promoting the synthesis of articular cartilage components. The specific mechanism(s) supporting cartilage regeneration remain to be elucidated. However, a growing body of research focusses on the paracrine and the immunoregulatory actions of MSCs. MSCs may stimulate cartilage regeneration by interacting with synovial macrophages, leading to a subsequent reduction in proinflammatory cytokines such as IL1β. Indeed, it was reported that MSCs administered into an osteoarthritic knee joint were in contact with synovial macrophages^8,50^, and were able to induce polarization towards M2 cells, which are involved in tissue repair^51–53^. In agreement with these findings, we also found evidence in the current study supporting the notion that IA-injected MSCs may decrease the inflammatory response caused by cartilage injury. However, further studies are required to elucidate the specific mechanisms.

The current work has some limitations. First, although our cell tracking system allows highly sensitive detection, it is not possible to obtain histological data from the whole tissue. Therefore, it cannot be totally excluded that further transgenic MSCs remained in distant organs or in knee joints for a longer period of time. Second, although our study addressed the biodistribution, engraftment, and efficacy of MSCs for regeneration of a focal cartilage defect, the specific pathways involved in the therapeutic effect remain elusive. It is clear that the molecular mode of action of MSC therapy needs to be addressed in future studies. Better insight into the mode of action of MSC therapy, and improved strategies to enhance local cell engraftment and survival may further enhance the therapeutic efficacy of this method.

## Methods

### Cytotoxicity and metabolic activity assays

MSCs were diluted to a final cell density of 10^7^ MSCs in 50 µL of native rat serum. A 1 mL syringe with a 23 G needle was used to deliver cells in a slow and steady injection into cell cultures dishes, mimicking the injections that were performed for intra-articular delivery of MSC *in vivo*. Post-ejection MSCs and non-ejected control MSCs were maintained in cell culture medium and incubated under hypoxic conditions. Analyses were performed 1 hour after ejection and at later time points (1, 2 and 5 days after ejection). The presence of lactate dehydrogenase (LDH) in culture media was used as an index of cell death. LDH activity was determined following the manufacturer’s instructions (Pierce LDH Cytotoxicity Assay Kit, Thermo Scientific, Rockford, USA). Cell metabolism was measured using PrestoBlue (Invitrogen, Carlsbad, CA, USA), following the manufacturer’s protocol. Data was normalized to the control group (100%).

### Animal model

The animal model used in this study is based on the human placental alkaline phosphatase, ALPP, as cell tracking marker. This enzyme is heat stable, but can be transformed into a heat-sensitive form by changing only one amino acid. Two different transgenic rat lines on inbred F344 genetic background were used for the experiment: a donor line expressing the heat-resistant protein (ALPP) and a recipient line expressing the heat-sensitive protein (ALPP^m^). Because these two protein forms differ in only a single amino acid, ALPP^m^-expressing recipient rats are tolerant to ALPP-labeled cells and tissues isolated from the transgenic ALPP donors^28^. All animals were housed in individually ventilated cages (Techniplast) at 24 °C and a 12 h/12 h light/dark cycle with free access to a standard rat chow and tap water. Two male rats were housed per cage.

### Focal cartilage defect

A focal full thickness articular cartilage defect was surgically created in the immunocompetent transgenic ALPP^m^ recipient rats. Briefly, cartilage defects were placed using a 0.7-mm NIH Style Neuro Punch (Fine Science Tools [FST]) in the patellofemoral groove of the left knee of male recipient animals at the age of 4–6 months under isoflurane anesthesia. The joint capsule and muscle tissue were sutured using 6-0 Vicryl (Ethicon), and the skin was closed using 4-0 Vicryl. For analgesia, oral metamizole (Novalgin™: 50 µL) was administered 30 min before and 6 h after surgery. Antibiosis was performed over 5 days after surgery using enrofloxacin (Baytril: 10 mg/kg s.c. once daily). The anti-inflammatory drug carprofen (Rimadyl: 20 µL s.c. once daily) was administered over 3 days after surgery in order to manage pain.

### Isolation and culture of BM-MSCs

Long bones including femora, tibiae and humeri were collected from 4- to 6-week-old ALPP-transgenic male rats for isolating bone marrow-derived mesenchymal stem cells (BM-MSCs). Animals were sacrificed by CO_2_ immediately before bone collection. Briefly, bones were cut at both epiphyses and incubated in cell culture medium containing collagenase. The bone marrow was flushed out, and Ficoll gradient centrifugation was performed for collecting the mononuclear cell fraction. Non-adherent cells were removed and MSCs were cultured under hypoxic conditions (37 °C, 5% CO_2_, and 5% O_2_) up to passage 5 as described previously^54^. Using this protocol, we reported in detail earlier that bone marrow-derived MSCs isolated from ALPP-transgenic donor rats maintained their immune phenotype as well as their differentiation capacity over six passages during *in vitro* cultivation^54^.

### IA injection of ALPP-labeled MSCs

For IA injection, 10^7^ transgenic donor MSCs were suspended in 50 µL of WT native rat serum isolated from whole blood. MSCs were intra-articularly injected using a 23 G needle into the knee cavity of transgenic ALPP^m^ recipients under isofluorane anesthesia, 2 weeks after defect creation. The knee joint was kept in a bent position, patella and femur were palpated, and ALPP-labeled MSCs were intra-articularly injected at a 90° angle from the medial side in the space between femur, tibia, and patella. Control animals were injected with rat serum, or left untreated. The rats (n = 4 per group and time point) were randomly allocated to the three groups, i.e., MSC-treated, serum control, and untreated. The body weight of the rats allocated to the treatment groups did not differ prior to treatment. For all experiments, the experimental unit was a single animal.

### Histological assessment

Heart, spleen, kidney, liver, and lung from ALPP^m^ recipient rats were collected 1 day, 1 week, 1 month, 2 months, and 6 months after injection of ALPP-labeled MSCs. Tissues were fixed in 40% ethanol and embedded in paraffin. Five-μm-thick sections on 3-aminopropyltriethoxy-silane (APES, Sigma) pretreated slides were prepared and 30–50 tissue sections per animal and organ were randomly selected from different tissue regions for further staining and evaluation. Briefly, samples were rehydrated, and heated at 72 °C for 1 h to block both endogenous ALP and ALPP^m^ activity. Sections were then incubated in TNM buffer (0.1 M Tris-HCl, pH 9.5, 0.1 M NaCl, 5 mM MgCl_2_) containing 0.17 μg/ml of the substrate 5-bromo-4-chloro-3-indolyl phosphate (BCIP; Sigma) and 0.45 μg/ml nitrotetrazolium blue chloride (NBT; Sigma) at room temperature overnight to detect ALPP activity. Subsequently, sections were counterstained with methyl green (Vector), dehydrated, and mounted using Vectamount (Vector).

Undecalcified dissected knee joints were directly embedded in frozen section compound (FSC 22; Leica) and snap-frozen in liquid nitrogen. Frozen samples were sectioned at 7 μm thickness on a Leica CM1950 cryostat with the help of an adhesive tape^55^. BCIP/NBT staining was performed as described above but reducing the heating time to 10 min in order to preserve tissue integrity in the cryosections. ALPP-positive area was quantified using ImageJ (NIH). Three different regions were identified and evaluated: synovial cavity, suprapatellar bursa and defect area. The total area as well as the corresponding ALPP-stained area in each region were measured and the presence of ALPP-labeled MSCs was given as percentage of total area. The defect site in every tissue section was identified with the help of the neotissue formed in the damaged area, as it is not exactly equal to the surrounding healthy cartilage. Knee joint cryosections were stained with toluidine blue (Sigma) and safranin O (Sigma) to evaluate the cartilaginous matrix. All histological analyses were performed in a non-blinded fashion.

### Immunofluorescence analysis

Knee cryosections were fixed with ice-cold ethanol and blocked with blocking buffer for 1 h at room temperature. The cryosections were incubated with primary antibodies against type I collagen (600-401-103, Rockland), type II collagen (CP18, Millipore), type X collagen (ab58632, Abcam), interleukin-1 beta (ab9722, Abcam), CD68 (MCA341R, BioRad), and ALPP (GTX72989, GeneTex or CBL207, Chemicon). Thereafter, sections were incubated with a mixture of FITC-conjugated anti-mouse (F0382, Sigma) and Alexa Fluor 555–conjugated anti-rabbit (4413, Cell Signaling) antibodies for 1 h at room temperature, air-dried, and mounted with Fluoromount (21644.01, Serva). DAPI (4083 S, Cell Signaling) was used to visualize nuclei. All immunofluorescence analyses were performed in a non-blinded fashion. Collagen stainings within the defect region were quantified as percent of defect area, using ImageJ.

### Statistical analysis

All data are presented as mean values ± standard error of the mean (SEM). Normal distribution of data was analyzed by Kolmogorov-Smirnov test. Differences between two independent groups were assessed using 2-tailed Student’s t-test. Data from more than two groups were analyzed using one-way analysis of variance (ANOVA) followed by Tukey’s multiple comparison test, or Kruskal-Wallis test followed by Mann-Whitney *U*-test, as appropriate (SPSS, Chicago, IL, USA). P < 0.05 was considered statistically significant.

### Study approval

All animal studies were approved by the Ethical Committees of the University of Veterinary Medicine, Vienna, and of the Austrian Federal Ministry of Science and Research, (permit no. BMWF-68.205/0146-II/3b/2013) and were undertaken in strict accordance with prevailing guidelines for animal care and welfare.

## Data Availability

All data generated or analysed during this study are included in this published article.
